# The ratio of serum C-reactive protein level on postoperative day 3 to day 2 is a good marker to predict postoperative complications after laparoscopic radical gastrectomy for gastric cancer

**DOI:** 10.1007/s00423-022-02469-w

**Published:** 2022-02-23

**Authors:** Bin Luo, Qianchao Liao, Jiabin Zheng, Weixian Hu, Xueqing Yao, Yong Li, Junjiang Wang

**Affiliations:** 1 Department of Gastrointestinal Surgery, Guangdong Provincial People’s Hospital, Guangdong Academy of Medical Sciences, No. 106, Zhongshan Er Road, Guangzhou, 510080 China; 2 Shantou University Medical College, No. 22, Xinling Road, Shantou, 515041 China

**Keywords:** C-reactive protein, Postoperative complications, Laparoscopic radical gastrectomy, Gastric cancer

## Abstract

**Purpose:**

Study reported that C-reactive protein (CRP) would peak at 48 h after the initiation of an acute inflammatory response. We proposed that the ratio of CRP level on postoperative day 3 to day 2 (POD3/2 CRP) can be used to early predict major postoperative complications (PCs) for patients who underwent laparoscopic radical gastrectomy.

**Methods:**

Patients were randomized into training cohort and validation cohort at a ratio of 7:3. PCs greater than grade II or more, according to Clavien-Dindo classification, were defined as major PCs. Three predictive models for major PCs based on CRP level were constructed, including POD3/2 CRP, the CRP level on POD3 (POD3 CRP), and the ratio of CRP level on POD3 to POD1 (POD3/1 CRP). The performances of three prediction models were assessed by AUC. Univariate and multivariate logistic regression analyses were performed to identify risk factors of major PCs.

**Results:**

344 patients were included. Major PCs were observed in 57 patients (16.6%). In the training cohort, POD3/2 CRP provided the best diagnostic accuracy with an AUC of 0.929 at an optimal cut-off value of 1.08, and the sensitivity and specificity were 0.902 and 0.880, respectively. In the validation cohort, the corresponding AUC was 0.917. BMI ≥ 25 kg/m^2^ and POD3/2 CRP > 1 were identified as risk factors for major PCs.

**Conclusion:**

POD3/2 CRP is a reliable marker to predict major PCs after laparoscopic radical gastrectomy. If CRP is higher on POD3 than on POD2, major PCs are highly likely.

**Supplementary Information:**

The online version contains supplementary material available at 10.1007/s00423-022-02469-w.

## Introduction

Gastric cancer (GC) is the fifth most common malignant tumor worldwide [1]. Even though significant improvements have achieved in surgical techniques and perioperative management, the morbidity rate after laparoscopic gastrectomy is still high [2–6]. Major postoperative complications (PCs) prolonged the hospitalization and increased mortality rate. It is reported that major PCs were also a risk factor of poor prognosis [7–10]. However, major PCs are often diagnosed after the patient develops severe clinical symptoms, which makes patient requires major clinical interventions such as intensive care and reoperation. Therefore, it is of great importance to diagnose major PCs at early time.

C-reactive protein (CRP) is an important systemic inflammatory marker. Elevated CRP level was ahead of the onset of descriptive clinical manifestation and positive imaging findings. There are several studies utilizing serum CRP level at a certain day to early predict major PCs for patients who underwent gastrectomy. However, the reported cut-off value varied greatly [11–15]. Study reported that CRP would peak at 48 h after the initiation of an acute inflammatory response [16]. So, we proposed that the ratio of CRP level on POD3 to day 2 (POD3/2 CRP) can be used to early predict major PCs for patients who underwent laparoscopic radical gastrectomy.

In this study, we aimed to investigate whether POD3/2 CRP can be used as an early predictor for major PCs, and to compare the diagnostic accuracy of POD3/2 CRP for major PCs with other reported predictive models.

## Materials and methods

### Patients and data

Patients who underwent laparoscopic radical gastrectomy for gastric cancer from January 2017 to December 2020 were included in this study. Patients with missing value for CRP levels on POD1 to POD3 were excluded from this study. Patients who suffered from infectious diseases with elevated CRP levels before surgery were also excluded. Patients’ data were retrieved from a prospectively maintained database which was updated by surgeon monthly, including age, gender, body mass index (BMI), preoperative serum albumin level, American Society of Anesthesiologists (ASA) score, neoadjuvant chemotherapy, TNM stage according to the 8th edition of AJCC/UICC classification for gastric cancer, type of resection, combined resection, operation time, blood loss, PCs, and postoperative hospitalization.

### Classification and diagnosis of postoperative complications

In this study, pneumonia, pleural effusion, anastomotic leakage, bleeding, duodenal stump leakage, abdominal abscess, ileus, chylous leakage, and surgical site infection were analyzed. The Clavien-Dindo classification was adopted for the classification of postoperative complications. Major PCs were defined as PCs of grade II or more. Patients with PCs of grade I, or who had no PCs, were classified into the minor/no PCs group. Pneumonia, pleural effusion, ileus, and abdominal abscess were confirmed by the computer tomography (CT) scan. Anastomotic leakage and duodenal stump leakage were diagnosed by CT and abnormal drainage.

### Surgical procedure and postoperative management

Most patients received D2 lymph node dissection. Additional mediastinal lymph nodes resection was performed for patients with Siewert type II adenocarcinoma of esophagogastric junction. Billroth II anastomosis was adopted for distal gastrectomy. Roux-en-Y anastomosis was carried out during total gastrectomy. As for proximal gastrectomy, double-tract anastomosis was the most common. The procedure of anastomosis was performed through laparoscopic approach or open approach. Patients who underwent distal gastrectomy were managed according to the enhanced recovery after surgery (ERAS) principle [17]. For patients who received total gastrectomy or proximal gastrectomy, oral diet was permitted only if esophageal dynamic radio-graphy demonstrated no evidence of anastomotic leakage on POD4 (Table 1).

**Table 1:** Detailed perioperative management procedure for patients who underwent laparoscopic radical gastrectomy

		**Distal gastrectomy**	**Total gastrectomy**	**Proximal gastrectomy**
**Gastrectomy-specific ERAS care**	Preoperative nutrition for malnourished patients	○	○	○
	Preoperative oral pharmaconutrion			
	Laparoscopic access	○	○	○
	Transversus abdominis plane block	○	○	○
	Nasogastric/nasojejunal decompression	○		
	Avoiding the use of abdominal drains			
	Early postoperative diet and artificial nutrition	○		
	Audit	○	○	○
**General ERAS care**	Dedicated preoperative counselling	○	○	○
	Abstinence of smoking and alcohol consumption	○	○	○
	Do not use mechanical bowel preparation	○	○	○
	Preoperative fasting and preoperative treatment with carbohydrates	○	○	○
	Optimal anaesthetic management	○	○	○
	Preanaesthetic medication			
	Antithrombotic prophylaxis	○	○	○
	Antimicrobial prophylaxis and skin preparation	○	○	○
	Epidural analgesia			
	Intravenous analgesia through PCA	○	○	○
	Multimodal intervention for PONV	○	○	○
	Avoiding intraoperative hypothermia	○	○	○
	Postoperative glycemic control	○	○	○
	Near-zero fluid balance	○	○	○
	Removing urinary drainage on POD1–2	○	○	○
	Stimulation of bowel movement	○	○	○
	Early and scheduled mobilization	○	○	○

*ERAS* enhanced recovery after surgery; *PCA* patient-controlled analgesia; *PONV* postoperative nausea and vomiting; *POD* postoperative day

### Statistical analysis

Patients were randomized into a training cohort and a validation cohort at a ratio of 7:3.

The Wilcoxon rank-sum test was used for non-normally distributed data. The *χ*^2^ test was performed to compare the enumeration data. The diagnostic accuracy of predictive models for major PCs was assessed by the area under the receiver operator curve (AUC). The optimal cut-off values were calculated by maximizing Youden’s index (sensitivity + specificity − 1). Univariate and multivariate logistic regressions were utilized to identify risk factors for postoperative complications. A two-side *p* value < 0.05 was considered significant. Statistical analysis was performed on SPSS (version 22.0 for Windows; SPSS Inc., Chicago, IL) and R software (version 4.0.3; http://www.r-project.org).

## Results

### Clinicopathologic characteristics

A total of 344 patients were enrolled in this study. Three hundred forty-four patients were randomized into a training cohort and a validation cohort at a ratio of 7:3.

As shown in Table 2, the training cohort was comprised of 164 males and 76 females with a median age of 64 (54.25–70) years. The median BMI was 22.15 (19.71–24.01) kg/m^2^, and the average preoperative serum albumin level was 37.71 ± 4.20 g/L (reference range: 40–55 g/L). The proportions of patients of stages I, II, and III were 32.9%, 24.2%, and 42.9%, respectively. 10.8% patients received neoadjuvant chemotherapy, and 2.9% patients underwent combined resection, including cholecystectomy, partial transverse colectomy, and splenectomy. 50.4% patients underwent laparoscopic total gastrectomy.

**Table 2:** Patients’ characteristics and differences between major complications group and minor/no complications group in training cohort

**Characteristics**	**Total**	**Major complications**^**a**^	**Minor/no complications**^**a**^	***p*** **value**
	***N*** **= 240 (%)**	***n*** **= 41 (%)**	***n*** **= 199 (%)**	
**Age**				
< 65 years	126 (52.5)	21 (51.2)	105 (52.8)	0.857
≥ 65 years	114 (47.5)	20 (48.8)	94 (47.2)	
**Gender**				
Male	164 (68.3)	30 (73.2)	134 (67.3)	0.296
Female	76 (31.7)	11 (26.8)	65 (32.7)	
**BMI (kg/m**^**2**^**)**	22.15 (19.71–24.01)	23.20 (21.78–26.15)	21.78 (19.38–23.70)	0.002^b^
**Preoperative serum albumin level (g/L)**	37.71 ± 4.20	38.51 ± 3.32	37.55 ± 4.35	0.719
**ASA score**				
I	26 (10.8)	4 (9.8)	22 (11.1)	0.949
II	207 (86.3)	36 (87.8)	171 (85.9)	
III	7 (2.9)	1 (2.4)	6 (3.0)	
**Neoadjuvant chemotherapy**				
Yes	26 (10.8)	4 (9.8)	22 (11.1)	1.000
No	214 (89.2)	37 (90.2)	177 (88.9)	
**TNM stage**				
I	79 (32.9)	16 (39.0)	63 (31.7)	0.143
II	58 (24.2)	5 (12.2)	53 (26.6)	
III	103 (42.9)	20 (48.8)	83 (41.7)	
**Type of resection**				
Distal gastrectomy	104 (43.3)	12 (29.3)	92 (46.2)	0.017
Total gastrectomy	121 (50.4)	23 (56.1)	98 (49.3)	
Proximal gastrectomy	15 (6.3)	6 (14.6)	9 (4.5)	
**Combined resection**				
Yes	7 (2.9)	1 (2.4)	6 (3.0)	1.000
No	233 (97.1)	40 (97.6)	193 (97.0)	
**Operation time (min)**	312.5 (255–312.5)	360 (295–442.5)	300 (250–340)	< 0.001^b^
**Blood loss (ml)**	50 (30–100)	100 (50–200)	50 (30–100)	0.010^b^
**Postoperative hospitalization (day)**	8 (6–10)	8 (6–9)	19 (11–37.5)	< 0.001^b^

*BMI* body mass index; *ASA* American Society of Anesthesiologists

^a^The major complication group was defined as patients with postoperative complications (PCs) of grade II or more according to the Clavien-Dindo classification. Patients with PCs of grade I, or who had no PCs, were classified into the minor/no PCs group

^b^Mann-Whitney test

The validation cohort consisted 67 males and 76 females with a median age of 61.5 (51.25–67) years. The median BMI was 21.62 (19.91–24.17) kg/m^2^, and the average preoperative serum albumin level was 38.25 ± 3.86 g/L. The proportions of patients of stages I, II, and III were 37.5%, 28.8%, and 33.7%, respectively. 11.5% patients received neoadjuvant chemotherapy. The rate of combined resection was 1.0%. About 50% patients underwent laparoscopic total gastrectomy (Table 3).

**Table 3:** Patients’ characteristics and differences between major complications group and minor/no complications group in validation cohort

**Characteristics**	**Total**	**Major complications**^**a**^	**Minor/no complications**^**a**^	***p*** **value**
	***N*** **= 104 (%)**	***n*** **= 16 (%)**	***n*** **= 88 (%)**	
**Age**				
< 65 years	63 (60.6)	8 (50.0)	55 (62.5)	0.347
≥ 65 years	41 (39.4)	8 (50.0)	33 (37.5)	
**Gender**				
Male	67 (64.4)	12 (75.0)	55 (62.5)	0.406
Female	37 (35.6)	4 (25.0)	33 (37.5)	
**BMI (kg/m**^**2**^**)**	21.62 (19.91–24.17)	22.12 (20.54–25.05)	21.48 (19.64–23.62)	0.136^b^
**Preoperative serum albumin level (g/L)**	38.25 ± 3.86	37.76 ± 3.61	38.34 ± 3.91	0.585
**ASA score**				
I	11 (10.6)	1 (6.3)	10 (11.4)	0.394
II	89 (85.6)	15 (93.7)	74 (84.1)	
III	4 (3.8)	0 (0.0)	4 (4.5)	
**Neoadjuvant chemotherapy**				
Yes	12 (11.5)	2 (12.5)	10 (11.4)	1.000
No	92 (88.5)	14 (87.5)	78 (88.6)	
**TNM stage**				
I	39 (37.5)	3 (18.8)	36 (40.9)	0.213
II	30 (28.8)	6 (37.5)	24 (27.3)	
III	35 (33.7)	7 (43.7)	28 (31.8)	
**Type of resection**				
Distal gastrectomy	45 (43.3)	4 (25.0)	41 (46.6)	0.090
Total gastrectomy	52 (50.0)	9 (56.3)	43 (48.9)	
Proximal gastrectomy	7 (6.7)	3 (18.7)	4 (4.5)	
**Combined resection**				
Yes	1 (1.0)	0 (0.0)	1 (1.1)	1.000
No	103 (99.0)	16 (100.0)	87 (98.9)	
**Operation time (min)**	300 (270–345)	345 (277.5–405)	300 (266.25–335)	0.030^b^
**Blood loss (ml)**	50 (50–100)	50 (50–150)	50 (50–100)	0.184^b^
**Postoperative hospitalization (day)**	8 (6–10)	29.5 (11.5–38.5)	7 (6–9)	< 0.001^b^

*BMI* body mass index; *ASA* American Society of Anesthesiologists

^a^The major complication group was defined as patients with postoperative complications (PCs) of grade II or more according to the Clavien-Dindo classification. Patients with PCs of grade I, or who had no PCs, were classified into the minor/no PCs group

^b^Mann-Whitney test

### Relationship between major PCs and clinical characteristics

As shown in Table 4, major PCs were observed in 57 patients (16.6%), including anastomotic leakage in 24 (7.0%), pneumonia and pleural effusion in 10 (2.9%), ileus in 7 (2.0%), and bleeding in 4 (1.2%).

**Table 4:** Information about major complications

Type of postoperative complications	No. (%)	Clavien-Dindo classification			
		II	III	IV	V
Anastomotic leakage	24 (7.0)	4	12	6	2
Pneumonia and pleural effusion	10 (2.9)	6	4		
Ileus	7 (2.0)	5	2		
Bleeding	4 (1.2)	1	2	1	
Duodenal stump leakage	2 (0.6)	1	1		
Abdominal abscess	4 (1.2)	1	3		
Chylous fistula	1 (0.3)	1			
Surgical site infection	1 (0.3)	1			
Other	4 (1.2)	1	2	1	
Total	57 (16.6)	21	26	8	2

In the training cohort, 41 patients developed major PCs and 199 patients had minor or no PCs. Patients in the major PCs group had higher BMI (23.20 kg/m^2^ vs. 21.78 kg/m^2^, *p* = 0.002), higher rate of total gastrectomy and proximal gastrectomy (70.7% vs. 53.8%, *p* = 0.017), longer operation time [360 (295–442.5) min vs. 300 (250–340) min, *p* < 0.001], and greater blood loss [100 (50–200) ml vs. 50 (30–100) ml, *p* = 0.010], than minor/no PCs group. There were no significant differences in age, gender, preoperative serum albumin level, ASA score, neoadjuvant chemotherapy, and TNM stage. Furthermore, the postoperative hospitalization of major PCs groups was significantly prolonged than minor/no PCs group [19 (11–37.5) days vs. 8 (6–9) days, *p* < 0.001] (Table 2).

In the validation cohort, 16 patients developed major PCs and 88 patients had minor or no PCs. Significant differences in BMI, operation time, and postoperative hospitalization were observed between the two groups. However, there were no significant differences in resection range and blood loss between the two groups.

### The variation tendency of serum CRP level

**Fig. 1:**
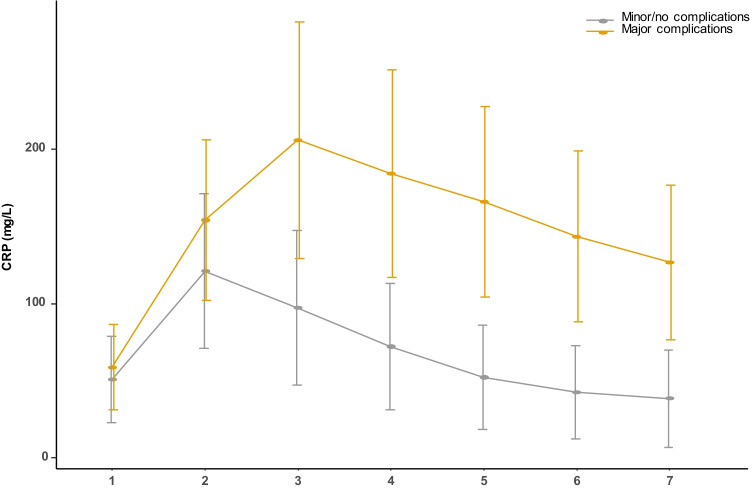
The variation tendency of serum CRP level in major complications group and minor/no complications group

As shown in Fig. 1, for patients with minor or no PCs, the serum CRP level peaked on POD2, and reduced to normal range, gradually. However, the serum CRP level for patients developed major PCs continued to increase on POD2 and maintained at a high level, even though effective antibiotics had been used.

### Diagnostic accuracy of different predictive models for major PCs

Based on above analysis, we proposed that POD3/2 CRP can be used to early predict major PCs.

**Fig. 2:**
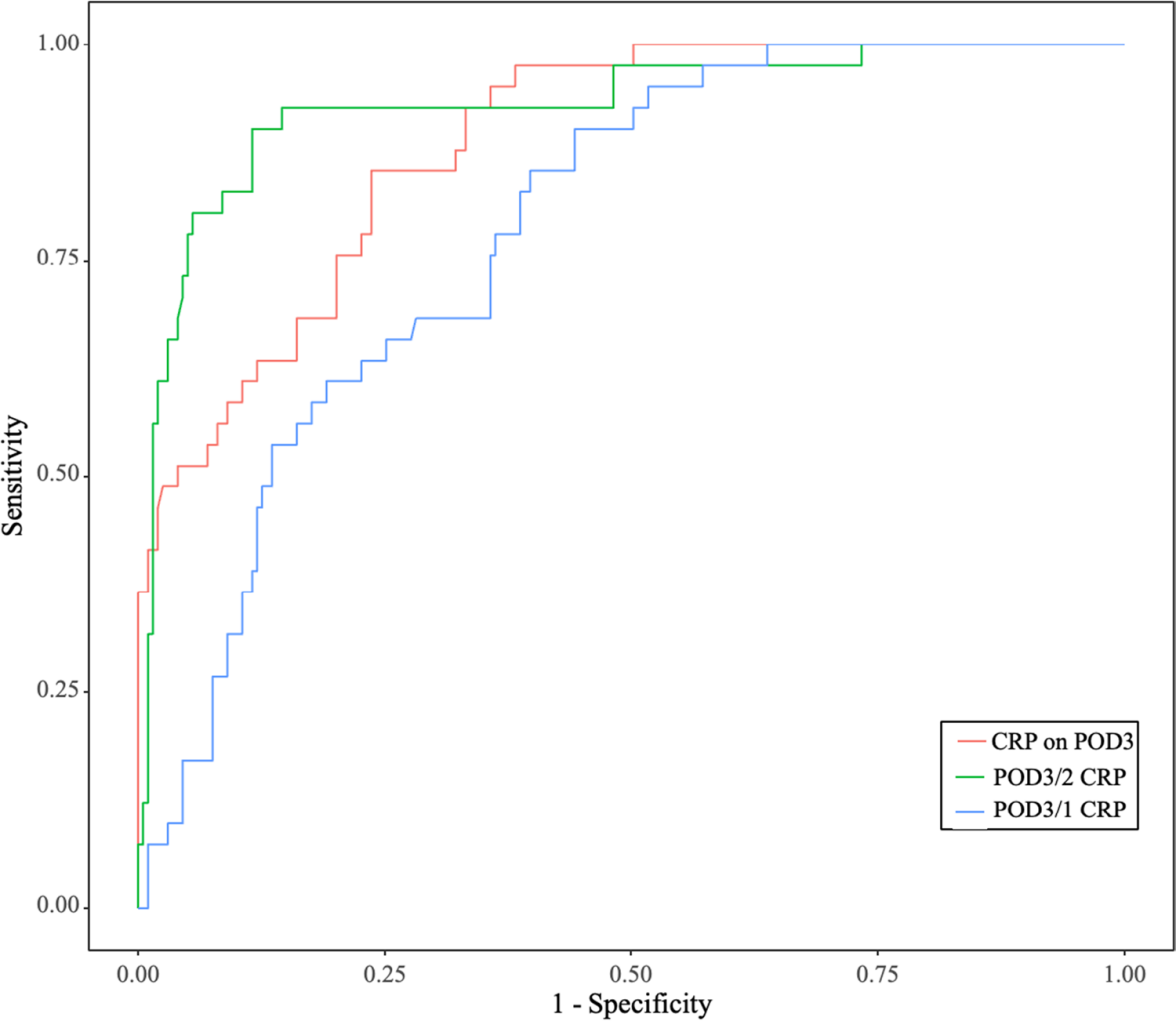
ROC curves for the diagnostic accuracy of prediction models based on CRP in training cohort

As shown in Fig. 2, in the training cohort, the AUC of POD3/2 CRP was 0.929, with an optimal cut-off value of 1.08, and the sensitivity and specificity were 0.902 and 0.880, respectively. The AUC of POD3 CRP was 0.886, with an optimal cut-off value of 128.1 mg/L. The corresponding sensitivity and specificity were 0.854 and 0.764, respectively. Another predictive model based on serum CRP level, POD3/1 CRP, was reported to be a good predictor for major PCs [18]. We utilized this model in the training cohort. The AUC of POD3/1 CRP was 0.786, with an optimal cut-off value of 1.890, and the sensitivity and specificity were 0.912 and 0.558, respectively (Table 5).

**Table 5:** Risk factors for major complications based on univariate and multivariate logistical regression analyses

**Characteristics**	**Univariate logistical analysis**			**Multivariate logistical analysis**		
	**OR**	**95% CI**	***p*** **value**	**OR**	**95% CI**	***p*** **value**
**Age**						
< 65 years	Reference					
≥ 65 years	1.216	0.688–2.149	0.500			
**Gender**						
Female	Reference			Reference		
Male	1.452	0.767–2.748	0.252	0.989	0.423–2.314	0.980
**BMI**						
< 25 kg/m^2^	Reference			Reference		
≥ 25 kg/m^2^	2.872	1.478–5.580	0.002	2.936	1.088–7.928	0.034
**Preoperative serum albumin level**						
≥ 40 g/L	Reference					
< 40 g/L	0.867	0.469–1.602	0.648			
**ASA score**			0.681			
I	Reference					
II	1.332	0.495–3.584	0.570			
III	0.640	0.067–6.142	0.699			
**Neoadjuvant chemotherapy**						
No	Reference					
Yes	0.891	0.373–2.358	0.891			
**T stage**						
T1–2	Reference					
T3–4	1.003	0.562–1.790	0.992			
**N stage**						
N0	Reference					
N1–3	1.259	0.706–2.244	0.435			
**Type of resection**			0.002			0.148
Distal gastrectomy	Reference			Reference		
Proximal gastrectomy	5.755	2.126–15.575	0.001	4.933	0.931–26.124	0.061
Total gastrectomy	1.887	0.990–3.596	0.054	1.309	0.430–3.982	0.636
**Combined resection**						
No	Reference					
Yes	0.714	0.086–5.920	0.755			
**Operation time**						
≤ 300 min	Reference			Reference		
> 300 min	3.009	1.614–5.608	0.001	2.108	0.706–6.293	0.181
**Blood loss**						
< 100 ml	Reference			Reference		
≥ 100 ml	1.751	0.988–3.105	0.055	0.958	0.374–2.452	0.928
**POD3/2 CRP**						
≤ 1.0	Reference					
> 1.0	37.422	15.971–87.687	< 0.001	42.548	16.967–106.700	< 0.001

*OR* odds ratio; *CI* confidence interval; *BMI* body mass index; *ASA* American Society of Anesthesiologists; *POD3/2 CRP* the ratio of CRP level on postoperative day 3 to day 2

**Fig. 3:**
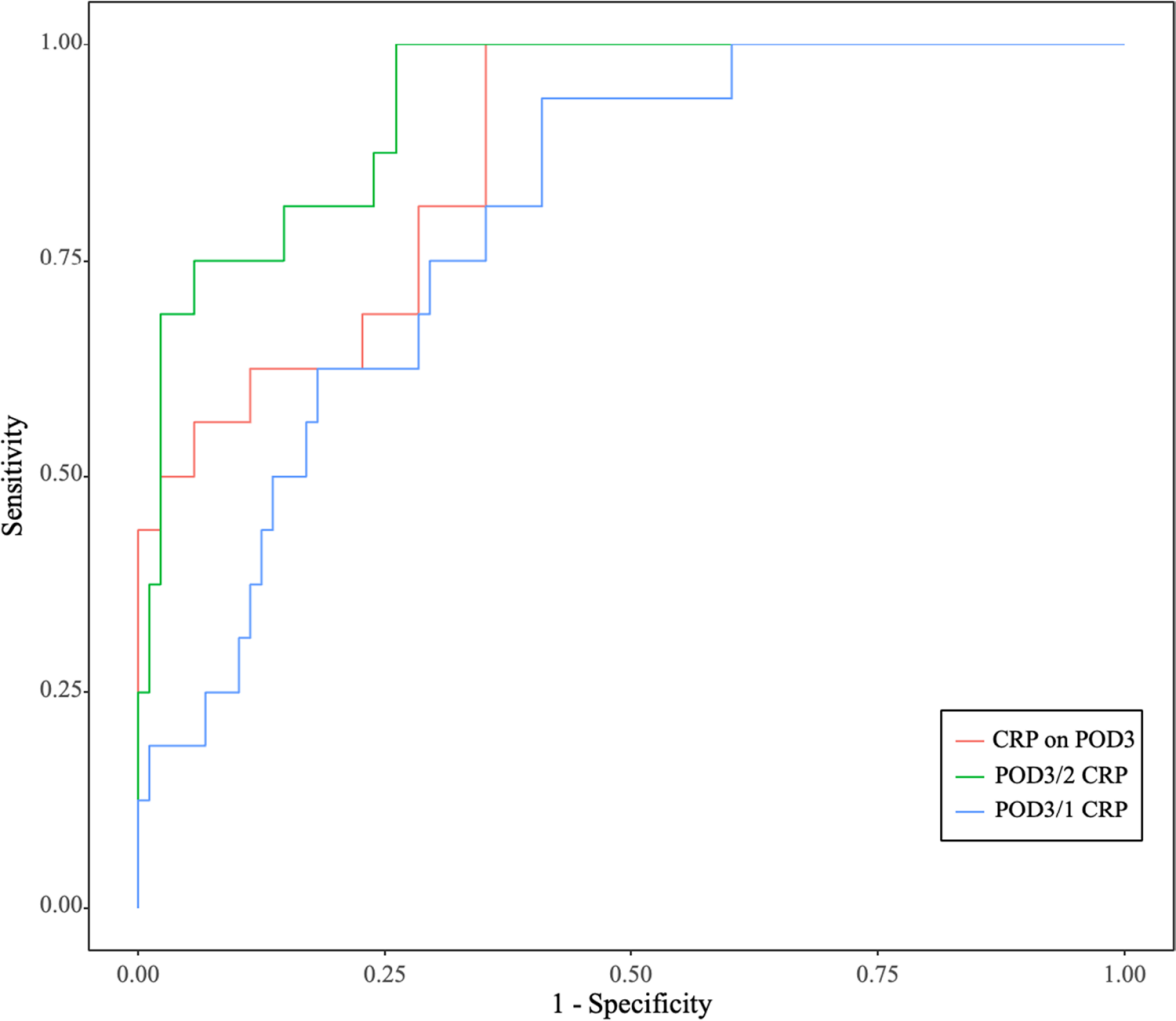
ROC curves for the diagnostic accuracy of prediction models based on CRP in validation cohort

In the validation cohort, the AUC of POD3/2 CRP was 0.917, the corresponding false positive rate and false negative rate were 14.8% and 18.8%, respectively. In detail, among the validation cohort of 104 patients, there were 26 patients whose POD3/2 CRP value was higher than the cut-off value. Of those, 13 patients developed major PCs, and 7 patients received over-diagnosis. The AUCs of POD3 CRP and POD3/1 CRP were 0.872 and 0.796, respectively (Fig. 3). In addition, we utilized reported cut-off values in the validation cohort and compared them with POD3/2 CRP approach, and we found that POD3/2 CRP provided the best Youden’s index (Table 6) [11–15, 18].

**Table 6:** Receiver operating characteristic analysis for the diagnosis of major PCs in validation cohort

Characteristics	Sensitivity	Specificity	Youden’s index
**Ratio**			
POD3/2 = 1.08	0.812	0.852	0.664
POD3/1 = 2.13 [18]	0.938	0.523	0.461
**Value**			
167 mg/L on POD5 [13]	0.455	1.000	0.455
168 mg/L on POD4 [14]	0.385	0.963	0.348
177 mg/L on POD3 [11]	0.563	0.909	0.472
114 mg/L on POD3 [12]	0.981	0.602	0.583
177 mg/L on POD2 [15]	0.438	0.830	0.268

### Risk factors analysis for major PCs

In the univariate logistic analysis, BMI ≥ 25 kg/m^2^ (OR = 2.872, 95% CI 1.478–5.580, *p* = 0.002), proximal gastrectomy (OR = 5.755, 95% CI 2.126–15.575, *p* = 0.001), operation time longer than 300 min (OR = 3.009, 95% CI 1.614–5.608, *p* = 0.001), and POD3/2 CRP > 1 (OR = 37.422, 95% CI 15.971–87.687, *p* < 0.001) were identified as risk factors for major PCs. Further analyzed by multivariate logistic analysis, BMI ≥ 25 kg/m^2^ and POD3/2 CRP > 1 were identified as risk factors for major PCs (Table 4).

## Discussion

In this study, we found that the serum CRP level peaked on POD2 in patients with minor or no PCs, and proposed that this feature could be used to early predict major PCs. Then, we compared the diagnostic accuracy of POD3/2 CRP with other reported predictive models, such as POD3 CRP and POD3/1 CRP, and found that POD3/2 CRP can provide the best performance in early predict major PCs with an optimal cut-off value of 1.08 (AUC = 0.929, sensitivity = 0.902, specificity = 0.880).

CRP was an acute-phase protein first reported in 1930 [19]. CRP was synthesized by hepatocytes quickly upon the inflammatory stimulation, and would peaked at 48 h after the initiation of an acute inflammatory response [16, 19]. This feature was consistent with our result that the mean CRP level in patients with no/minor PCs peaked at POD2 and reduced to baseline gradually. Another study, comparing the differences in CRP level for patients who underwent emergency or elective colorectal surgery, also reported this feature [20]. Other studies investigating the value of CRP level in early predicting major PCs did not detect CRP level on POD2 routinely. We recommended that serum CRP level should be tested routinely on POD 1, 2, and 3, and then examined according to patients’ status. In addition, the variation tendency of serum CRP level is a reliable marker to indicate the presence of major PCs.

There were several studies using the cut-off value of CRP at a certain day to early predict the onset of PC [11–15]. As a systematic inflammatory factor, serum CRP level varied individually according to age, sex, nutrient status, and operation [21–24]. Therefore, the diagnostic accuracy of postoperative serum CRP level on a certain day was not very precise. The reported cut-off value of CRP level varied greatly. Shishido et al. found that CRP level on POD3 had the highest diagnostic accuracy for PCs with a cut-off value of 177 mg/L [11]. The optimal cut-off value of CRP level on POD3 reported by Okubo et al. was 114 mg/L [12]. Utilizing the variation tendency of CRP for early prediction of major PCs can avoid above limitation caused by individual heterogeneity. Tanaka et al. used POD3/1 CRP to predict the onset of PCs [18]. In their study, they reported a cut-off CRP ratio of 2.13 with 55% sensitivity and 82% specificity for major PCs. In our study, we also constructed the POD3/1 CRP model. We found that POD3/1 CRP had slight superiority in sensitivity (0.912 vs. 0.902), but significant shortage in specificity (0.558 vs. 0.880), compared with POD3/2 CRP. This means patients would receive extra examinations if treatment strategy was made based on POD3/1 CRP model.

Some studies tried to use the CRP level on POD5 to increase the specificity and negative predictive value [13, 25]. However, with the increasing popularity of ERAS, more and more patients underwent laparoscopic distal gastrectomy discharged on POD4. This method is not suitable for hospitals which have rich experience in ERAS because of short postoperative hospitalization.

The multivariate analysis suggested that BMI ≥ 25 kg/m^2^ significantly increased the risk of major PCs (OR 2.936, 95% CI 1.088–7.928, *p* = 0.034). Because extended lymph node dissection may be hampered by excess bodyweight [26–28], this finding was consistent with other studies [29, 30]. Interestingly, our research revealed that proximal gastrectomy with double-tract anastomosis (PG-DTR) may increase the risk of major PCs (OR 4.933, 95% CI 0.931–26.124, *p* = 0.061). Compared with Roux-en-Y reconstruction for total gastrectomy, one more anastomosis, gastrojejunostomy, is performed. This procedure prolongs the operation time and double anastomotic stoma means double risk of leakage. However, PG-DTR was considered superior to total gastrectomy with Roux-en-Y reconstruction in terms of nutrition [31]. Hence, PG-DTR procedure may be performed by experienced surgeon in not fat patients.

The limitations of this study included its retrospective and single-institution design. Prospective studies should be performed to investigate whether early diagnostic or therapeutic approaches based on POD3/2 CRP could actually lead to earlier detection of infectious complications and improve outcomes.

## Conclusion

POD3/2 CRP is a reliable marker to predict major PCs after laparoscopic radical gastrectomy. If CRP is higher on POD3 than on POD2, major PCs are highly likely.

BMI ≥ 25 kg/m^2^ and POD3/2 CRP >1 were identified as significant independent risk factors for major PCs.

## Supplementary Information

ESM 1 (XLSX 46 kb)

ESM 2 (XLSX 25 kb)

## Data Availability

The raw data supporting the conclusions of this article will be made available by the authors, without undue reservation.

## References

[1] Bray F, Ferlay J, Soerjomataram I (2018). Global cancer statistics 2018: GLOBOCAN estimates of incidence and mortality worldwide for 36 cancers in 185 countries. CA Cancer J Clin.

[2] Liu F, Huang C, Xu Z (2020). Morbidity and mortality of laparoscopic vs open total gastrectomy for clinical stage I gastric cancer: the CLASS02 multicenter randomized clinical trial. Jama Oncol.

[3] Lee HJ, Hyung WJ, Yang HK (2019). Short-term outcomes of a multicenter randomized controlled trial comparing laparoscopic distal gastrectomy with D2 lymphadenectomy to open distal gastrectomy for locally advanced gastric cancer (KLASS-02-RCT). Ann Surg.

[4] Li Z, Shan F, Ying X (2019). Assessment of laparoscopic distal gastrectomy after neoadjuvant chemotherapy for locally advanced gastric cancer: a randomized clinical trial. Jama Surg.

[5] Kim W, Kim HH, Han SU (2016). Decreased morbidity of laparoscopic distal gastrectomy compared with open distal gastrectomy for stage I gastric cancer: short-term outcomes from a multicenter randomized controlled trial (KLASS-01). Ann Surg.

[6] Chen QY, Xie JW, Zhong Q (2020). Safety and efficacy of indocyanine green tracer-guided lymph node dissection during laparoscopic radical gastrectomy in patients with gastric cancer: a randomized clinical trial. Jama Surg.

[7] Tokunaga M, Tanizawa Y, Bando E (2013). Poor survival rate in patients with postoperative intra-abdominal infectious complications following curative gastrectomy for gastric cancer. Ann Surg Oncol.

[8] Krarup PM, Nordholm-Carstensen A, Jorgensen LN (2014). Anastomotic leak increases distant recurrence and long-term mortality after curative resection for colonic cancer: a nationwide cohort study. Ann Surg.

[9] Nathan H, Yin H, Wong SL (2017). Postoperative complications and long-term survival after complex cancer resection. Ann Surg Oncol.

[10] Saunders JH, Yanni F, Dorrington MS (2020). Impact of postoperative complications on disease recurrence and long-term survival following oesophagogastric cancer resection. Br J Surg.

[11] Shishido Y, Fujitani K, Yamamoto K (2016). C-reactive protein on postoperative day 3 as a predictor of infectious complications following gastric cancer resection. Gastric Cancer.

[12] Okubo K, Arigami T, Matsushita D (2021). Clinical impact of creatine phosphokinase and c-reactive protein as predictors of postgastrectomy complications in patients with gastric cancer. BMC Cancer.

[13] Shi J, Wu Z, Wang Q (2020). Clinical predictive efficacy of C-reactive protein for diagnosing infectious complications after gastric surgery. Ther Adv Gastroenterol.

[14] Kim EY, Yim HW, Park CH (2017). C-reactive protein can be an early predictor of postoperative complications after gastrectomy for gastric cancer. Surg Endosc.

[15] Ji L, Wang T, Tian L (2016). The early diagnostic value of C-reactive protein for anastomotic leakage post radical gastrectomy for esophagogastric junction carcinoma: a retrospective study of 97 patients. Int J Surg.

[16] Gabay C, Kushner I (1999). Acute-phase proteins and other systemic responses to inflammation. N Engl J Med.

[17] Mortensen K, Nilsson M, Slim K (2014). Consensus guidelines for enhanced recovery after gastrectomy: Enhanced Recovery After Surgery (ERAS®) Society recommendations. Br J Surg.

[18] Tanaka H, Tamura T, Toyokawa T (2019). C-reactive protein elevation ratio as an early predictor of postoperative severe complications after laparoscopic gastrectomy for gastric cancer: a retrospective study. BMC Surg.

[19] Tillett WS, Francis T (1930). Serological reactions in pneumonia with a non-protein somatic fraction of pneumococcus. J Exp Med.

[20] Straatman J, de Weerdesteijn EW, Tuynman JB (2016). C-reactive protein as a marker for postoperative complications. Are there differences in emergency and elective colorectal surgery?. Dis Colon Rectum.

[21] Kuczmarski MF, Mason MA, Allegro D (2013). Diet quality is inversely associated with C-reactive protein levels in urban, low-income African-American and white adults. J Acad Nutr Diet.

[22] Ahonen TM, Kautiainen HJ, Keinanen-Kiukaanniemi SM (2008). Gender difference among smoking, adiponectin, and high-sensitivity C-reactive protein. Am J Prev Med.

[23] Khera A, McGuire DK, Murphy SA (2005). Race and gender differences in C-reactive protein levels. J Am Coll Cardiol.

[24] Brooks GC, Blaha MJ, Blumenthal RS (2010). Relation of C-reactive protein to abdominal adiposity. Am J Cardiol.

[25] Park JH, Son YG, Kim TH (2017). Identification of candidates for early discharge after gastrectomy. Ann Surg Oncol.

[26] Kodera Y, Ito S, Yamamura Y (2004). Obesity and outcome of distal gastrectomy with D2 lymphadenectomy for carcinoma. Hepatogastroenterology.

[27] Dhar DK, Kubota H, Tachibana M (2000). Body mass index determines the success of lymph node dissection and predicts the outcome of gastric carcinoma patients. Oncology.

[28] Inagawa S, Adachi S, Oda T (2000). Effect of fat volume on postoperative complications and survival rate after D2 dissection for gastric cancer. Gastric Cancer.

[29] Xiao H, Pan SG, Yin B (2013). Clavien-Dindo classification and risk factors for complications after radical gastrectomy for gastric cancer. Zhonghua Yi Xue Za Zhi.

[30] Kodera Y, Sasako M, Yamamoto S (2005). Identification of risk factors for the development of complications following extended and superextended lymphadenectomies for gastric cancer. Br J Surg.

[31] Kim DJ, Kim W (2016). Laparoscopy-assisted proximal gastrectomy with double tract anastomosis is beneficial for vitamin B12 and iron absorption. Anticancer Res.

